# Supplementary material for: Retrieval of the Complete Coding Sequence of the UK-Endemic Tatenale Orthohantavirus Reveals Extensive Strain Variation and Supports Its Classification as a Novel Species
Source: Viruses. 2020 Apr 17;12(4):454. doi: 10.3390/v12040454 (PMC7232349; doi:10.3390/v12040454)
Supplement: Supplementary file 1 [file viruses-12-00454-s001.pdf]

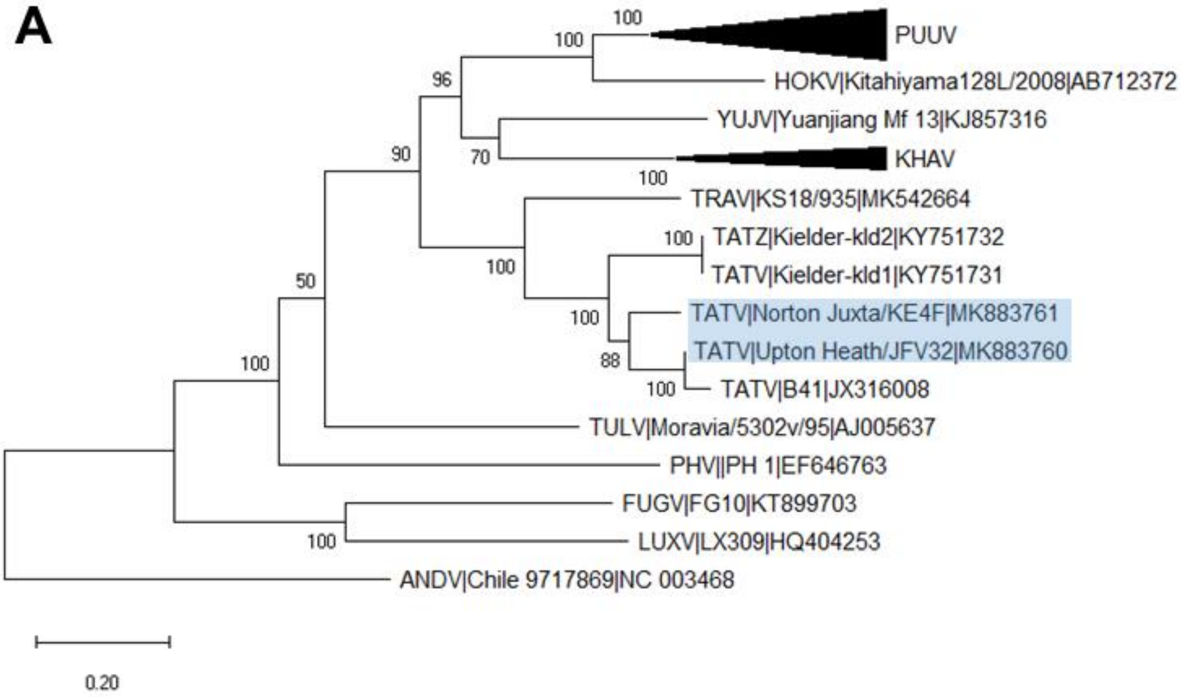
A
100
100
PUUV
96
HOKV|Kitahiyama128L/2008|AB712372
YUJV|Yuanjiang Mf 13|KJ857316
90
70
KHAV
100
TRAV|KS18/935|MK542664
100
TATZ|Kielder-kld2|KY751732
100
TATV|Kielder-kld1|KY751731
50
100
TATV|Norton Juxta/KE4F|MK883761
100
88
TATV|Upton Heath/JFV32|MK883760
100
TATV|B41|JX316008
TULV|Moravia/5302v/95|AJ005637
PHV||PH 1|EF646763
FUGV|FG10|KT899703
100
LUXV|LX309|HQ404253
ANDV|Chile 9717869|NC 003468
0.20

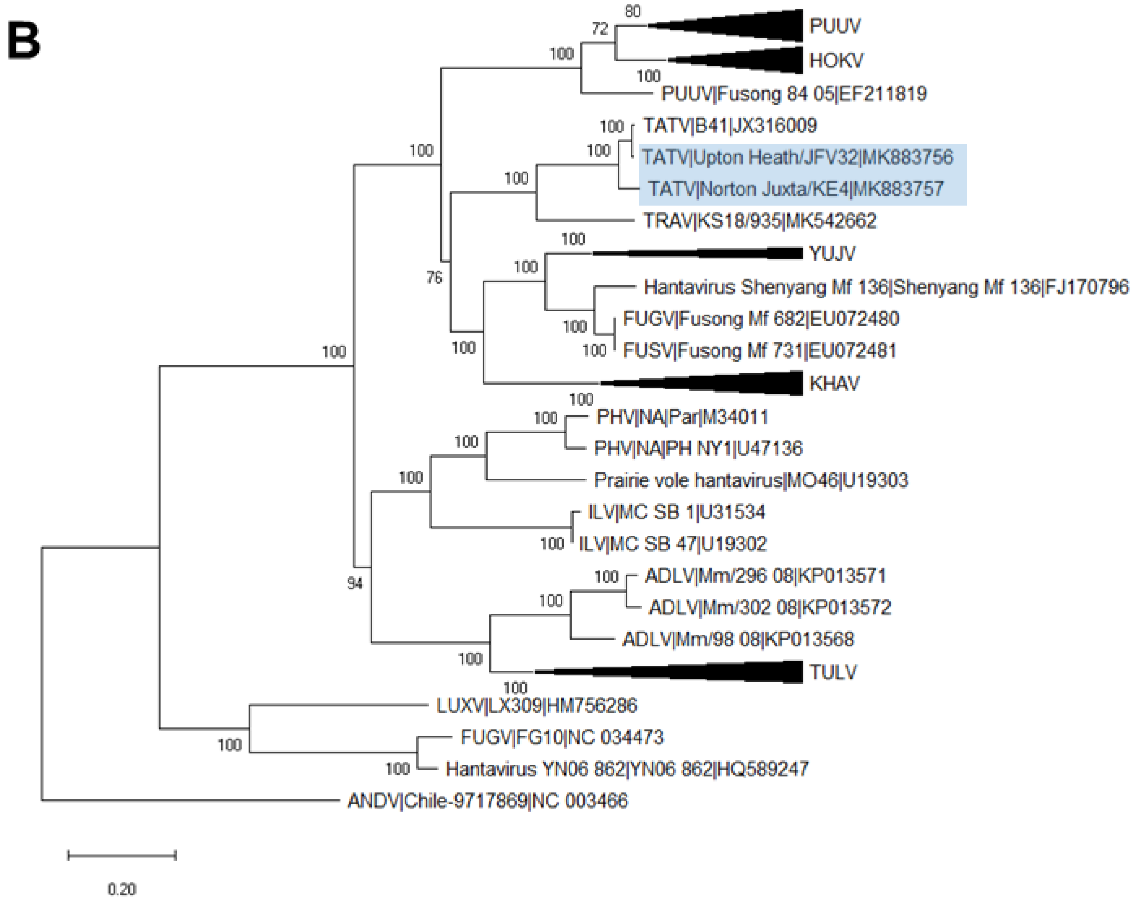

Supplementary Figure 1: Phylogenetic relationship of Tatenale virus Upton-Heath and Norton-Juxta, with available partial sequences of previous Tatenale virus sequences and other vole-associated orthohantavirus species. Representative partial sequences were obtained for L (A) and S (B) segments. Maximum Likelihood trees were created using the GTR+G+I model, branch lengths are drawn to a scale of nucleotide substitutions per site. Numbers above individual branches show bootstrap support after 50 replicates. Tatenale virus strains discovered in this study are highlighted with a blue box. Sequences are shown with the species name, strain name and the GenBank accession number. Black triangles represent a compressed species, specific subtree. PUUV, Puumala virus; HOKV, Hokkaido virus; FUSV, Fusong virus; YUJV, Yuanjiang virus; KHAV, Khabarovsk virus; TATV, Tatenale virus; PHV, Prospect Hill virus; ILV, Isla Vista virus; TULV, Tula virus; ADLV, Adler virus; LUXV, Luxi virus; FUGV, Fugong virus; TRAV, Traemmersee virus.
